# Numerical Investigation of Masonry Strengthened with Composites

**DOI:** 10.3390/polym10030334

**Published:** 2018-03-19

**Authors:** Giancarlo Ramaglia, Gian Piero Lignola, Francesco Fabbrocino, Andrea Prota

**Affiliations:** 1 Department of Structures for Engineering and Architecture, University of Naples Federico II, Via Claudio 21, 80125 Naples, Italy; giancarlo.ramaglia@unina.it (G.R.); aprota@unina.it (A.P.); 2 Department of Engineering, Telematic University Pegaso, Piazza Trieste e Trento, 48, 80132 Naples, Italy; francesco.fabbrocino@unipegaso.it

**Keywords:** masonry, strengthening system, basalt, hemp, curvature ductility, numerical investigation

## Abstract

In this work, two main fiber strengthening systems typically applied in masonry structures have been investigated: composites made of basalt and hemp fibers, coupled with inorganic matrix. Starting from the experimental results on composites, the out-of-plane behavior of the strengthened masonry was assessed according to several numerical analyses. In a first step, the ultimate behavior was assessed in terms of P (axial load)-M (bending moment) domain (i.e., failure surface), changing several mechanical parameters. In order to assess the ductility capacity of the strengthened masonry elements, the P-M domain was estimated starting from the bending moment-curvature diagrams. Key information about the impact of several mechanical parameters on both the capacity and the ductility was considered. Furthermore, the numerical analyses allow the assessment of the efficiency of the strengthening system, changing the main mechanical properties. Basalt fibers had lower efficiency when applied to weak masonry. In this case, the elastic properties of the masonry did not influence the structural behavior under a no tension assumption for the masonry. Conversely, their impact became non-negligible, especially for higher values of the compressive strength of the masonry. The stress-strain curve used to model the composite impacted the flexural strength. Natural fibers provided similar outcomes, but a first difference regards the higher mechanical compatibility of the strengthening system with the substrate. In this case, the ultimate condition is due to the failure mode of the composite. The stress-strain curves used to model the strengthening system are crucial in the ductility estimation of the strengthened masonry. However, the behavior of the composite strongly influences the curvature ductility in the case of higher compressive strength for masonry. The numerical results discussed in this paper provide the base to develop normalized capacity models able to provide important information on the out-of-plane behavior of masonry elements strengthened with inorganic matrix and several kinds of fibers, both synthetic and natural.

## 1. Introduction

A large part of the existing building stock is made of masonry structures, and recent calamitous events have shown the high vulnerability of these structures. The strong vulnerability is due to different factors: many structures were built in periods when the lack of building codes promoted design approaches based on empirical and geometrical rules. Furthermore, masonry structures are often characterized by a strong heterogeneity both in the materials and in the building techniques. Tight measures were applied only for particular prestigious buildings. In fact, many heritage buildings showed high seismic performances under several earthquakes [1]. Conversely, a large part of the ordinary building stock was built according to poor construction techniques. In these cases, without specific measures, most of the ordinary building stock shows a high seismic vulnerability. Many failure modes are due to local mechanisms rather than global mechanisms. In this background, the modelling of masonry structures represents a crucial aspect. The issues increase when the attention is focused on poorly built structures. Recent scientific research on innovative materials confirmed that the modern strengthening techniques are very effective in improving the seismic behavior of existing structures. In the strengthening field, several materials can be used for existing structures. The present work focuses on novel composite materials made of inorganic matrices and synthetic or natural fibers. Basalt fibers guarantee high mechanical compatibility with good masonry (i.e., solid clay brick) if compared to other fibers like carbon, because of the lower elastic modulus (closer to masonry modulus). Similarly, hemp fibers show high mechanical compatibility with poor masonries (i.e., adobe masonry), because the elastic modulus is lower for both. Furthermore, these composites can be easily applied in heritage buildings where the restoration criteria must be satisfied.

Hemp fibers provide low environmental impact on the built environment. Low impact now represents a primary condition to be respected by the construction industry. Natural fibers represent a highly sustainable material, and there is increasing scientific interest in construction applications, due to their good mechanical properties. In fact, the tensile strength of fibers can reach even 1000 MPa in the case of flax fiber, and about 800 MPa in the case of jute fiber, whereas hemp fiber exhibits a tensile strength up to 690 MPa. The Young’s modulus is about 30 GPa for jute and flax fiber, while for hemp fiber it varies up to 60 GPa. However, woven composites exhibit lower performance, but natural fibers can be competitive with the most-used synthetic fibers for masonry applications. Recent studies provide an overview of the different natural fiber composites available in the market [2,3].

The numerical results discussed in this paper provide important information on the out-of-plane behavior of masonry elements strengthened with inorganic matrix and several kinds of fibers. They constitute the base to develop normalized capacity models, which are currently not available in the building codes.

Many engineering applications have proved the potential of composite materials made of synthetic fibers (carbon or glass) and organic matrix (resin). Carbon fiber reinforced polymers (CFRPs) and glass fiber reinforced polymers (GFRPs) have been widely used in many applications on different kinds of masonry [4,5,6]. However, for some masonries these strengthening techniques may show some problems (e.g., the presence of mortar joints limiting the bond performance [7], the need to improve the bond [8], or the dynamic response [9]). In addition to experimental activity, Finite Element Modelling (FEM) for brick masonry [10] or adobe [11] has also been used to deepen knowledge on strengthening different kinds of masonry. In particular, some materials like resins and carbon could be incompatible with the restoration criteria—especially when the intervention strategies involve heritage buildings. Therefore, inorganic materials usually replace the resin, such as lime or cementitious-based mortar to bond carbon [12], basalt [13], steel [14], or glass [15] fibers to masonry. These composites are named fabric-reinforced cementitious matrix (FRCM) or textile-reinforced mortar (TRM), having a higher physical and mechanical compatibility with masonry substrate [16,17,18]. This property refers both to the fiber and to the matrix. In fact, the resins show several issues when applied on damp substrates such as the masonry. Similarly, basalt or hemp show mechanical properties more compatible with the substrate. High-performance fibers are less efficient due to the high differences between composite and substrate. Furthermore, previous issues increase when the strengthening interventions are performed on weak masonries like adobe [11], and in this case, highly performing materials like basalt would not allow the full exploitation of the strengthening system’s capacity. Fiber elements made of natural materials would ensure greater compatibility with the weak masonry substrate [2,3]. Furthermore, in poor regions, the low cost of natural fibers makes them a good alternative to the classical synthetic systems, given their several advantages, such as recycling, reduced pollution, and ready availability. These strengthening systems certainly satisfy the sustainability requirements. 

The positive effects of the previously discussed strengthening systems have been demonstrated in many scientific works [19,20,21,22,23]. Great confirmations have been provided by the experimental activity [19,20], but also by the numerical activity [21,22,23]. The common experimental approach is based on statically- or dynamically-tested masonry specimens before and after the strengthening intervention. However, without a normalized design approach, many intervention strategies are characterized by low efficiency. With reference to the strengthening strategies applied on concrete or steel structures, several analytical models are available and many of them were included in modern building codes.

In many engineering applications, the interventions on masonry in the past were performed without a proper design, or simply according to suggestions by manufactures. This approach often favors low efficiency of the strengthening systems or a deleterious behavior of composites. In particular, composites applied in unnecessary amount present low internal stresses (low efficiency), and they could favor a brittle behavior of the masonry elements, pushing the failure of the masonry.

Given the previously discussed background, the structural analyses in this study focused on composites made of inorganic matrix and natural (hemp) and synthetic (basalt) fibers. The experimental results allow the two composites to be numerically modeled. A solver algorithm was implemented to parametrically assess the ultimate behavior of strengthened masonry elements, changing several parameters. In a first step, the ultimate behavior was assessed in terms of the P (axial load)-M (bending moment) failure surface.

The maximum bending moment of the strengthened masonry element was evaluated starting from the bending moment-curvature diagram. This approach allows the internal stresses of the composite to be monitored up to the ultimate state of the materials. Furthermore, this approach allows the assessment of the impact of several parameters on the entire load curve. In fact, as clarified in the following section, some parameters which apparently do not affect the maximum bending moment greatly alter the entire bending moment-curvature diagram. This represents a critical aspect in order to perform non-linear analyses. In particular, the bending moment-curvature diagram provides important information on the ductility capacity of the masonry elements strengthened with composites.

For this reason, the bending moment-curvature diagrams were bi-linearized according to several approaches to estimate the impact of key parameters on the ductility capacity of the masonry elements strengthened with composite materials. The numerical results allow the behavior of composites to be assessed under different load conditions. Furthermore, key information about the efficiency and the failure modes of the strengthening system and masonry system were obtained. They constitute the basis of developing normalized capacity models for structural nonlinear analyses of masonry structures strengthened with composite materials. This study is mainly oriented to analyze the flexural behavior, neglecting shear and flexure/shear interaction, as is common, for instance, in curved structures (e.g., arches and barrel vaults [24,25]).

## 2. Theoretical Approach

Several tests were performed to provide both the entire stress-strain curves and the load capacity by means of tensile tests on FRCM [2,12,13,14,15]. Starting from test results, the constitutive models of composites were derived to perform the numerical investigation. The great number of tested specimens allows to consider an average behavior of the masonry elements strengthened with composite: inorganic matrix and synthetic (basalt) or natural (hemp) fibers.

The failure modes of the tested specimens can be due to the rupture of the fiber or the delamination. In the first case, the tensile strength of the fiber is achieved after the matrix cracking. The latter failure mode occurs for substrates characterized by poor adhesion properties. 

Experimental results allow to identify different conventional threshold behaviors for the strengthening systems. In particular, the stress-strain curve starts with an almost linear response. It depends on the composite systems (combination of fiber and matrix) [26]. Under low stress level, both the fiber and the matrix carry the load and the initial curve presents a stiffness that depends mainly on the Young’s modulus of the matrix *E_mx_*. The initial almost linear stress-strain curve ends when the matrix cracks. In this case, the stiffness reduces, but it is higher than the Young’s modulus of the fiber, *E_f_*. In fact, after the cracking, the stress-strain curve depends on the tension stiffening phenomena. In particular, the matrix cracks according to a non-uniform distribution [27,28]. The cracks develop in a finite number of sections and, where the matrix is cracked, the load is carried by the fibers only. Conversely, between cracks the internal stresses can be transferred by means of bond from the fiber to the matrix. Therefore, the stiffness of the curve gradually shifts to the fibers only behavior with a progressive reduction of the stiffness to *E_f_*. This effect influences the entire response of the composite and depends on the mechanical and physical characteristics of the constituent materials.

When the fiber element is made of basalt grid, according to experimental results [13], two conventional behaviors can be identified. For a very weak matrix, the tension stiffening phenomena tend to zero and the transfer from the non-cracked to the cracked condition occurs at very low stress levels. For this reason, the entire stress-strain curve mainly involves the behavior of the fiber element only (this is very similar to the behavior of FRP).

When the composite is made of high performance matrices, the cracking threshold occurs at high stress levels [28,29,30]. In this case, once the cracking threshold is achieved, the stress-strain curve progressively shifts to the curve of the fiber element only. According to the experimental results, the previous behavior typically characterizes the composite material made of inorganic matrix and basalt grid [13].

When natural fiber (hemp) replaces synthetic elements, the behavior changes. The hemp fiber is characterized by lower mechanical properties if compared to the synthetic fibers. In this background, using highly performing mortars is often useless [4]. Therefore, also the matrix must be made of materials compatible with the natural fiber and the masonry substrate. For these composites, the shift from the non-cracked to the cracked condition is always present. Therefore, if the matrix is made of extremely weak materials, stress-strain curve almost immediately shifts from the uncracked to the cracked condition. Conversely, if the matrix is characterized by more compatible properties with the fiber, the stress-strain curve shifts from the uncracked to the cracked condition progressively. According to the experimental results, the latter is typical of composite systems made of inorganic matrix and hemp fibers [27].

### 2.1. Mechanical Models of Materials

Strengthened masonry is made of materials characterized by different mechanical properties. These materials can be modelled by using several approaches [31]. In this work, several strategies were used to model the strengthened system and the masonry. The masonry was assumed to carry loads both in tension (to some extent) and in compression according to different mechanical behaviors, while the strengthening system has strict no-compression behavior, carrying tensile stresses only.

The masonry in compression was modelled according to the ideal mechanical model of Eurocode 6 [32]. Initially, the curve is governed by a parabolic function. It depends on the first plastic strain *ε*_0_ conventionally fixed equal to 2‰ and on the compressive strength, *σ*_0_. For strains higher than *ε*_0_, the stress is constantly equal to *σ*_0_ up to the maximum compressive strain *ε_mu_*, conventionally fixed equal to 3.5‰. These limit strains are provided by Eurocode 6. This model is certainly simplified and similar to models for concrete, as in both cases, experimental tests show similar stress-strain curves, with softening after the peak load [33,34]. More complex stress-strain models can be adopted, but the Eurocode 6 model was preferred to have a more general result.

Conversely, a brittle model was adopted for masonry in tension—in particular, a linear stress-strain curve up to the low tensile strength of masonry, *σ_t_*, with a drop to zero afterwards. It is assumed that portions of masonry in tension can be cracked, hence they do not carry loads: this allows assessment of the cracking and the effect of some masonry parameters (Young’s modulus and tensile strength) on the ultimate behavior of the strengthened masonry element.

When the strengthening system is included, the effect of the tensile strength of masonry on flexural capacity becomes negligible; however, the tensile strength usually has an impact on the entire response of the strengthened masonry, and hence in global nonlinear analyses of strengthened masonry structures.

Several approaches can be used to numerically model an experimental stress-strain curve. The multilinear model appears to be the favorite approach to modeling an experimental stress-strain curve, which can be easily implemented into solver algorithms. However, the available experimental results support an even more simplified approach. In fact, the strengthening system can be modelled by linear, bi-linear, or tri-linear relationships.

For strengthening systems made of inorganic matrix and basalt fiber, the experimental results confirm the linear and the bi-linear trends [12,13,14,15]. Instead, when natural hemp fiber replaces the synthetic fiber, the experimental results confirm the bi-linear and tri-linear trends [2,3].

In order to implement previous mechanical models into a solver algorithm, a homogenized approach can be suitable. The entire stress-strain curves can be homogenized to the fiber or to the matrix, meaning that the global behavior of the FRCM is attributed to one of the two constituents, by means of fictitious (i.e., homogenized) equivalent properties. The experimental tests provide the ultimate strain of composite *ε_c,u_* and the tensile strength *σ_c,u_*. According to the typical failure modes of composites, these values generally correspond to those of fiber *ε_f,u_* and the tensile strength *σ_f,u_*. From these values, a Young’s modulus of the fiber only can be calculated as *E_f_* = *σ_f,u_*/*ε_f,u_*. From the experimental tests, the cracking level can be assessed from the stress-strain curve. As previously discussed, a representative average behavior was chosen to perform the numerical modelling. 

In particular, the tensile strength of mortar *σ_m,cr_* provides the stress level at the cracking threshold. The associated strain level, *ε_m,cr_*, provides the cracking strain of composite *ε_c,cr_*, assuming a perfect bond between fiber and matrix. In the same way, the Young’s modulus of the matrix can be estimated as *E_mx_* = *σ_m,cr_*/*ε_m,cr_*. Starting from these values, the stress-strain diagram—referred to the fiber element—can be assessed. In particular, the initial homogenized (i.e., referred to fiber cross-section) modulus *E*_1_^*^ can be calculated as follows:

(1) $$E_{1}^{*}=\frac{E_{f}\cdot t_{f}+E_{mx}\cdot t_{m}}{t_{f}}$$

where *t_f_* is the thickness of the fiber and *t_m_* is the thickness of the matrix. Once the initial Young’s modulus *E*_1_^*^ is known, the cracking homogenized stress level can be evaluated as σ_m,cr_^*^ = *E*_1_^*^·*ε_m,cr_*.

The homogenized mechanical parameters allow the simplified stress-strain curve of the composite to be numerically modeled, according to the experimental results [2,12,13,14,15]. The linear model fits the behavior of the fiber only, starting from a zero strain up to the ultimate strain of the composite *ε_f,u_* (linear stress-strain diagram with stiffness equal to *E_f_*). Conversely, the bi-linear model is made of two main parts. A first linear part with slope *E*_1_^*^ ranging between zero strain up to cracking strain *ε_c,cr_* and a second linear diagram characterized by a stiffness *E*_2_^*^ higher than *E_f_*:

(2) $$E_{2}^{*}=\frac{\sigma_{c,u}-\sigma_{c,cr}}{\varepsilon_{c,u}-\varepsilon_{c,cr}}$$

According to a tri-linear model, the stress of the composite increases up to the cracking value σ_m,cr_^*^ (cracking strength of mortar homogenized to fiber). At increasing strain *ε* > *ε_c,cr_*, the stress remains constantly equal to σ_m,cr_^*^ until the strain reaches the value *ε*_2_ = σ_m,cr_^*^/*E_f_*. When the tensile stress increases, the matrix cracks and the fibers only carry loads in the strengthening system. For this reason, the stress-strain curve of composites close to failure tends to overlap with the fiber-only behavior (e.g., the stiffness of the composite is similar to the stiffness of the fiber).

The mechanical models described previously were used to perform the numerical investigation. These simplified stress-strain diagrams can be considered representative of an average behavior of limit behaviors of these composites.

### 2.2. Bi-Linearization of the Bending Moment-Curvature Diagram

The numerical approach discussed in Appendix A provides the non-linear bending moment-curvature diagrams. In order to implement these diagrams into global numerical nonlinear analyses of masonry structures, it can be useful to simplify them. Non-linear analyses are usually performed according to bi-linearized simplified models of the bending moment-curvature diagrams (elastic-perfectly plastic behavior).

Several bi-linearization approaches exist in the scientific literature [35]. Many of them are based on energetic criteria. According to energy-based criteria, the bi-linear curve is defined guaranteeing the same area of the original non-linear curve. Furthermore, the bi-linearized approach allows the assessment of the ductility capacity of the masonry element. Two main approaches can be used to bi-linearize the nonlinear diagrams. A first approach fixes the maximum bending moment and changes the stiffness of the initial linear curve (i.e., elastic-linear behavior) to match the two areas under the curves. This bi-linearized curve provides the minimum ductility capacity; it represents the best approach if the interest focuses on the ductility estimation, but could provide low estimates of the initial stiffness. 

As an alternative, a second approach fixes the inclination of the initial curve through a particular intersection point between the nonlinear and the bi-linear curves. This point is generally fixed at 60% or 70% of the maximum load value. According to this second approach, the energetic criterion is satisfied changing the maximum bending moment. The bi-linearized curve obtained with the latter approach provides a ductility capacity higher than the previous one, but it provides a better estimate of the stiffness of the curve. In this numerical investigation, the first approach was adopted because the main interest is in (safe side) ductility estimations of the strengthened masonry elements.

## 3. Numerical Investigation

### 3.1. Mechanical Characteristics of Materials

The previously discussed numerical model was applied to perform a numerical investigation on masonry elements strengthened with composite systems. Two strengthening systems were chosen to perform the parametric investigation:

- Composite made of inorganic matrix and basalt fiber (synthetic fiber);
- Composite made of inorganic matrix and hemp fiber (natural fiber).

**Figure 1:**
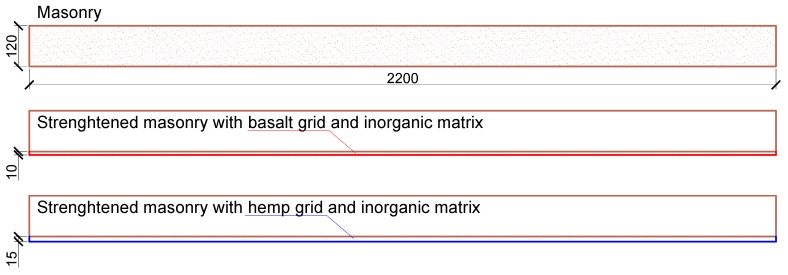
Main geometrical characteristics of masonry cross-section (dimensions in mm).

The geometry of the masonry refers to a masonry barrel vault actually tested at the University of Naples, Federico II [36,37]. In particular, the cross-section vault has dimensions b × s (width × height) equal to 2200 × 120 mm^2^. Masonry barrel vault can be analyzed as an arch with a slender cross-section when it is loaded in its mid-plane [36,37]. A single layer of composite was applied on one side. In many engineering applications, the strengthening system covers the elements on one side only, for several reasons (i.e., presence of decorations, mosaics, paintings, etc.). The numerical investigation refers to these cases. However, the strengthening system is supposed to be effective in tension only, as in compression the contribution is neglected due to potential instability issues [29,37]. The thickness of the matrix is equal to 10 and 15 mm for the synthetic and natural fibers, respectively. The geometrical characteristics are shown in Figure 1.

A bi-directional grid was used for both strengthening systems. The basalt grid had cross-section equal to 39.09 mm^2^/m (i.e., equivalent thickness of 0.039 mm). The hemp grid was made of strands characterized by an average diameter of 3.0 mm and a spacing of 20 mm, which corresponds to a cross-section of 353.25 mm^2^/m (i.e., equivalent thickness of 0.353 mm).

**Figure 2:**
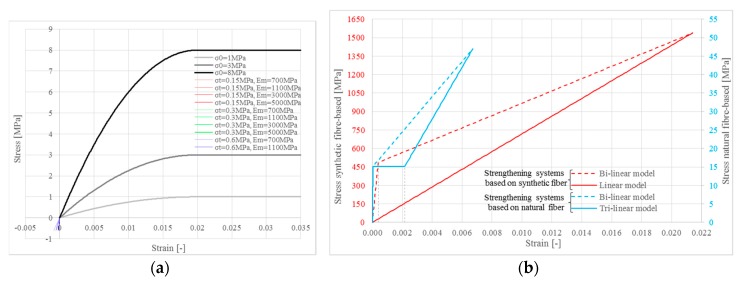
Stress-strain curves: (**a**) masonry material; (**b**) strengthening systems based on synthetic fiber (red lines) and based on natural fiber (blue lines).

The mechanical properties of masonry change according to the masonry typologies found in the Italian Building Code [38], (Figure 2a); however, for extremely weak masonry, the tensile strength *σ_t_* and the Young’s modulus *E_m_* were limited, as shown in Table 1. In particular, for a compressive strength of 1 MPa, the tensile strength was limited to 0.3 MPa and the Young’s modulus to 1100 MPa, as typically occurs in weak masonry. The weaker masonry represents a typical masonry made of tuff stone and weak mortar, while the upper bound properties are representative of a masonry made of clay bricks and cementitious mortar according to the Italian Building Code [38].

With reference to the strengthening systems, the mechanical properties change both for the matrix and for the fiber element. The mechanical properties of single elements are shown in Table 2. Table 3 shows the mechanical properties of the stress-strain curve homogenized to the fiber section (some values seem high, but they refer to fibers only, hence stresses become forces through multiplication by thin fiber cross-sections). These values refer to an average behavior of the two strengthening systems, as reported in data sheets [39,40]. They were used to perform the numerical investigation (Figure 2b).

Figure 2 shows the numerical stress-strain curves used to perform the numerical investigation for the masonry (Figure 2a) and for the strengthening systems (Figure 2b). Masonry curves in tension are close to each other.

More than 2000 numerical tests were performed combining the mechanical models used for the materials.

### 3.2. P-M Domains: Assessment of Masonry Cross-Sections Strengthened with Composite

**Figure 3:**
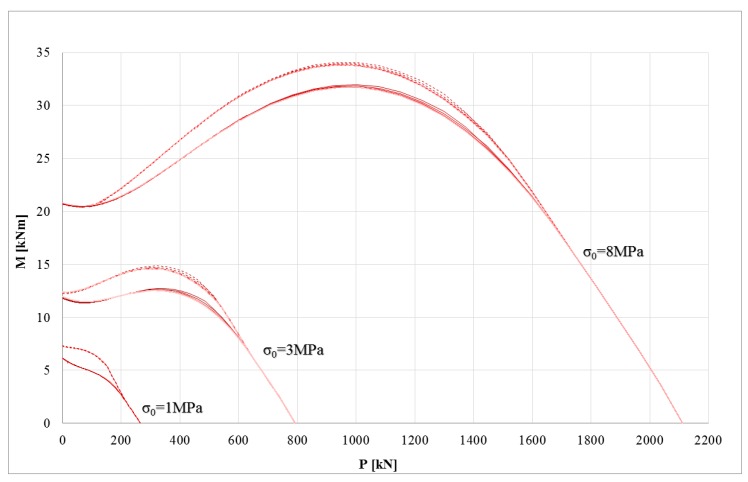
P (axial load)-M (bending moment) domains of the strengthening system with synthetic fiber: bi-linear model (dotted line), linear model (solid line).

**Figure 4:**
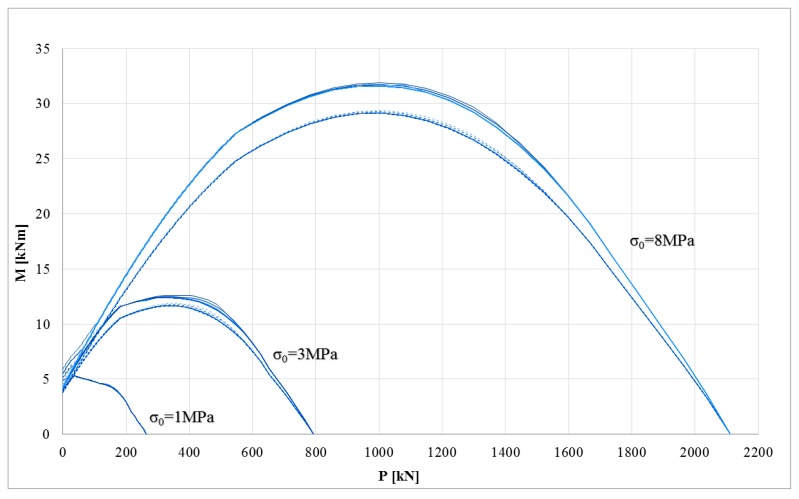
P-M domains of the strengthening system with natural fiber: bi-linear model (dotted line), tri-linear model (solid line).

The first goal of this research regards the assessment of the impact of mechanical parameters on the ultimate performance of masonry cross-sections strengthened with composites. The symmetry of the failure surface is missing because the strengthening system was applied on one side only. The mechanical properties of masonry provide a negligible influence on the maximum bending moment. This effect is due to the sharp difference between masonry and strengthening system at ultimate conditions. In fact, when the strengthening systems carry loads (i.e., under tensile stress), the Young’s modulus and tensile strength of the masonry show a negligible influence. This effect is shown for all the stress-strain models of the composites and for both strengthening systems (synthetic and natural fibers). Conversely, the constitutive laws used to model the composite provide significant differences in the P-M failure surface. Figure 3 shows the P-M domains for a masonry cross-section strengthened with composite made of inorganic matrix and synthetic fiber (basal grid), while Figure 4 shows the counterpart with natural fiber (hemp grid). In Figure 3 and Figure 4, the numerical results of the linear, bi-linear, and tri-linear models used for the strengthening systems are shown with different lines (dotted and solid, respectively) according to the corresponding constitutive law depicted in Figure 2b. The three different masonry constitutive laws (different Young’s modulus and tensile strength for each compressive strength *σ*_0_) provide negligible effects; in fact, the three corresponding curves are almost overlapping (e.g., the three dotted lines are almost overlapping).

It is interesting to note that the impact of the stress-strain model used for the composite prevails for masonry with lower mechanical properties. In particular, for low compressive strength masonry, the stress-strain model of the composite has a strong impact on the P-M domain, particularly at low axial load levels. This effect decreases upon increasing the compressive strength of the masonry. In fact, for weak masonry, the ultimate condition is generally due to the failure of the masonry. However, the impact of the stress-strain model of the FRCM decreases in the case of hemp because the mechanical properties of the materials (masonry and composite) become closer.

At very high axial loads, the curves are perfectly overlapping because the strengthening system is not loaded there and the cross-section is almost entirely compressed.

### 3.3. Bending Moment-Curvature Diagrams

When the focus is on the ultimate behavior of the strengthened masonry, the Young’s modulus and the tensile strength *σ_t_* apparently do not affect the load-carrying capacity of the masonry cross-section strengthened with composites [41]. However, it is interesting to assess the entire bending moment-curvature diagrams upon changing several parameters.

**Figure 5:**
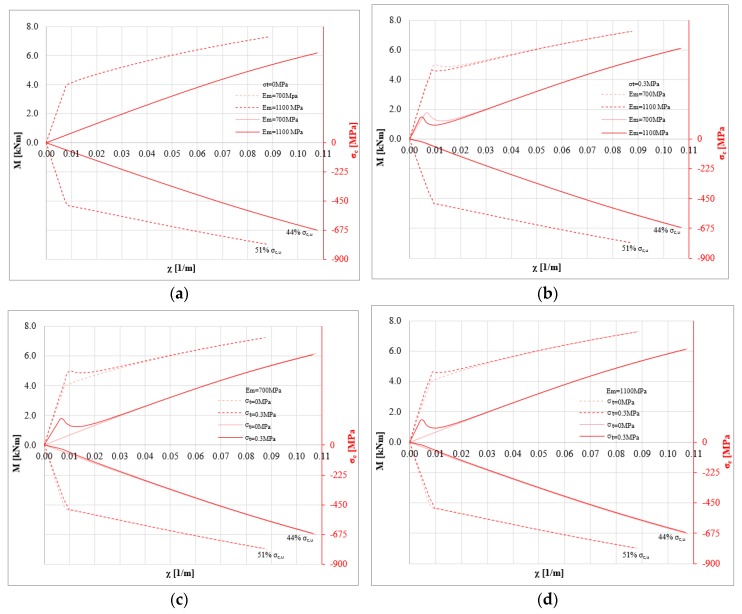
M-*χ* and *σ_c,u_*-*χ* diagrams: *P* = 0 N, *σ*_0_ = 1 MPa, *σ_t_* = 0 and 0.3 MPa, *E_m_* = 700 and 1100 MPa for linear (solid lines) and bi-linear (dotted lines) relationships for the strengthening system (basalt fiber): (**a**) *σ*_0_ = 1 MPa, *σ_t_* = 0 MPa changing *E_m_* (**b**) *σ*_0_ = 1 MPa, *σ_t_* = 0.3 MPa to changing *E_m_* (**c**) *σ*_0_ = 1 MPa, *E_m_* = 700 MPa changing *σ_t_* (**d**) *σ*_0_ = 1 MPa, *E_m_* = 1100 MPa changing *σ_t_*.

Figure 5 shows the bending moment-curvature diagrams (left black axis) and the corresponding stress-strain curve of the strengthening system (red axis) when parametrically changing the tensile strength and the Young’s modulus of masonry. The diagrams were assessed for a null value of the axial load *P*, where the numerical differences are more clear. Furthermore, the stress level achieved by the composite system is shown (as percentage of composite tensile strength). This information is crucial to assess the efficiency of the strengthening system. It refers to the masonry element strengthened with inorganic matrix and basalt grid. Figure 5a,b show the cases with compressive strength *σ*_0_ of masonry equal to 1 MPa. Furthermore, the two stress-strain curves to model the composite are shown: linear (solid line) and bi-linear (dotted line) model.

When no tension is assumed, the Young’s modulus *E_m_* provides negligible impact on the P-M domains. In this case, the several P-M domains are overlapping and this parameter provides no influence on the bending moment-curvature diagram. Figure 5a shows the strong difference in terms of the maximum bending moment between the two stress-strain curves of the composite. In these cases, the efficiency reduces when considering a linear model for the composite (about 40% and 50% for the linear and bi-linear, respectively). Assuming a tensile strength for the masonry (Figure 5b), the bending moment-curvature diagram depends on the Young’s modulus of the masonry. In particular, upon increasing the Young’s modulus, the cracking point becomes clear on the curve. Furthermore, the efficiency of the composite decreases when the Young’s modulus increases.

Figure 5c,d show the impact of the tensile strength *σ_t_* on the global behavior of the strengthened masonry. The tensile strength of masonry provides an increase of the initial stiffness and a small decrease of the efficiency on the composite. This effect is shown for both mechanical models—bi-linear (dotted lines) and linear (solid lines)—used to model the composite. The impact of the tensile strength of masonry *σ_t_* for the linear model is higher than for the bi-linear model. When tensile strength *σ_t_* is assumed for masonry, the bending moment-curvature curve presents a cusp, and the Young’s modulus has an influence on them, due to the cracking behavior. Increasing the axial load *P* (or the normalized axial load *p* = *P*/*P*_0_), the Young’s modulus has less influence on the bending moment-curvature diagram.

Another key parameter is represented by the stress level achieved in the strengthening system. When basalt fiber is applied on weak masonry substrates, its stress level remains low. In all cases, the stress level is lower than 50% of the tensile strength of the composite. This result confirms that the strengthening amount is frequently excessive.

**Figure 6:**
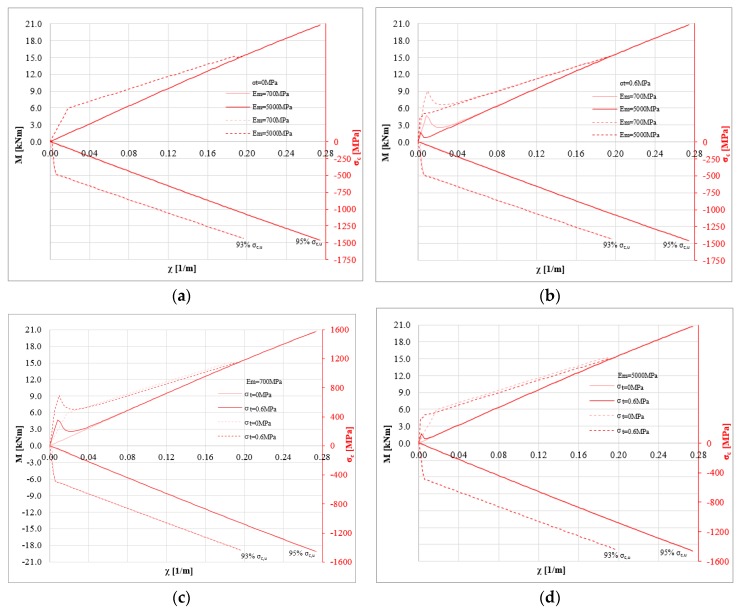
M-*χ* and *σ_c,u_* diagrams: *P* = 0 N, *σ*_0_ = 8 MPa, *σ_t_* = 0 and 0.6 MPa, *E_m_* = 700 and 5000 MPa for linear (solid lines) and bi-linear (dotted lines) relationships for the strengthening system (basalt fiber): (**a**) *σ*_0_ = 8 MPa, *σ_t_* = 0 MPa changing *E_m_* (**b**) *σ*_0_ = 8 MPa, *σ_t_* = 0.6 MPa to changing *E_m_* (**c**) *σ*_0_ = 8 MPa, *E_m_* = 700 MPa changing *σ_t_* (**d**) *σ*_0_ = 8 MPa, *E_m_* = 5000 MPa changing *σ_t_*.

Figure 6 shows the same numerical results on masonry characterized by a compressive strength *σ*_0_ = 8 MPa. Figure 6a,b show the influence of the Young’s modulus on the bending moment-curvature diagrams. The impact of Young’s modulus is clear, given the higher compressive strength of the masonry. This allows the stress level of the strengthening system to be increased, increasing its efficiency. In particular, for several numerical analyses, the stress level of the composite reaches about 90% of the ultimate strength. When the tensile strength is zero, the impact of the Young’s modulus is negligible. However, as evident from recent studies, no tension should not be assumed to estimate the load capacity of some masonry structures [37]. Finally, the compressive strength of masonry has a significant influence on the efficiency of the strengthening system.

**Figure 7:**
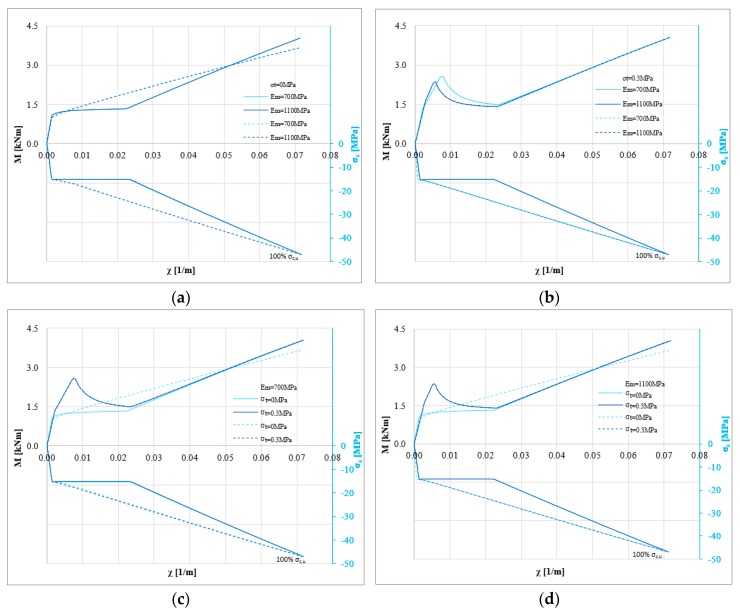
M-*χ* and *σ_c,u_*-*χ* diagrams: *P* = 0 N, *σ*_0_ = 1 MPa, *σ_t_* = 0 and 0.3 MPa, *E_m_* = 700 and 1100 MPa for tri-linear (solid lines) and bi-linear (dotted lines) relationships for the strengthening system (hemp fiber): (**a**) *σ*_0_ = 1 MPa, *σ_t_* = 0 MPa changing *E_m_* (**b**) *σ*_0_ = 1 MPa, *σ_t_* = 0.3 MPa to changing *E_m_* (**c**) *σ*_0_ = 1 MPa, *E_m_* = 700 MPa changing *σ_t_* (**d**) *σ*_0_ = 1 MPa, *E_m_* = 1100 MPa changing *σ_t_*.

A similar approach was used for the composite made of inorganic matrix and natural fiber (hemp fiber). In this case, bi-linear and tri-linear constitutive laws were adopted, according to the experimental results. Figure 7 shows the bending moment-curvature diagrams for masonry with compressive strength *σ*_0_ equal to 1 MPa under pure flexure (i.e., without axial load P). Figure 7a,b show the impact of the Young’s modulus for the two values of tensile strength of masonry, while Figure 7c,d show the influence of the tensile strength. The higher mechanical compatibility of the composite with the masonry substrate guarantees a high efficiency of the retrofit strategy. In fact, for all the numerical analyses, the failure of the strengthening system is reached. When the strengthening system becomes more compatible with the substrate, the effect of the elastic properties of masonry on the ultimate behavior becomes clearer. In these cases, the Young’s modulus and the tensile strength of masonry have a clear impact on the behavior of the strengthened masonry. In particular, assuming a tensile strength, the cracking behavior could govern the capacity. Furthermore, when the strengthening system becomes more compatible with the masonry, the maximum bending moment of the strengthened masonry cross-section is not strongly influenced by the stress-strain curve of the strengthening system. However, it can strongly influence the ductility of the masonry cross-section, as clarified in the following.

**Figure 8:**
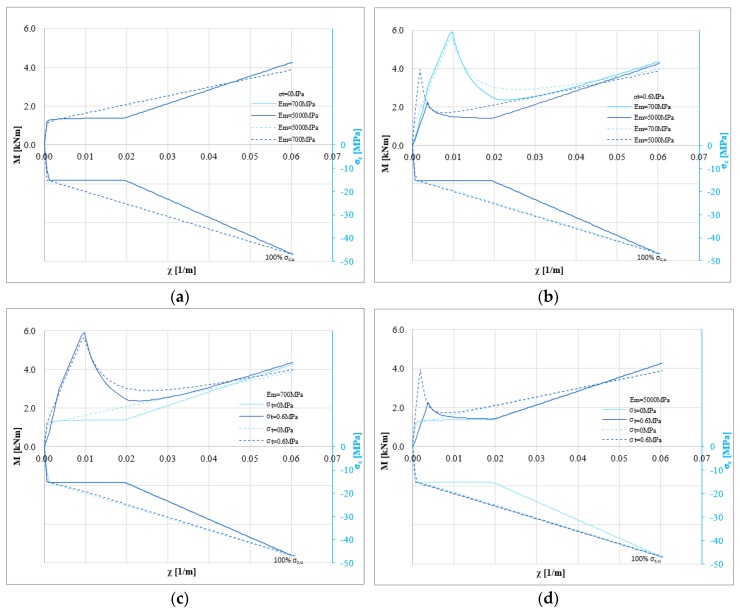
M-*χ* and *σ_c,u_*-*χ* diagrams: *P* = 0 N, *σ*_0_ = 8 MPa, *σ_t_* = 0 and 0.6 MPa, *E_m_* = 700 and 5000 MPa for tri-linear (solid lines) and bi-linear (dotted lines) relationships for the strengthening system (hemp fiber): (**a**) *σ*_0_ = 8 MPa, *σ_t_* = 0 MPa changing *E_m_* (**b**) *σ*_0_ = 8 MPa, *σ_t_* = 0.6 MPa to changing *E_m_* (**c**) *σ*_0_ = 8 MPa, *E_m_* = 700 MPa changing *σ_t_* (**d**) *σ*_0_ = 8 MPa, *E_m_* = 5000 MPa changing *σ_t_*.

Figure 8a–d outline the same results on masonry characterized by compressive strength *σ_m_* equal to 8 MPa. In this case, the cracking behavior is clear and it can control the capacity. In fact, the cusp limits the ductility, promoting a brittle failure mode.

### 3.4. Ductility Estimation

**Figure 9:**
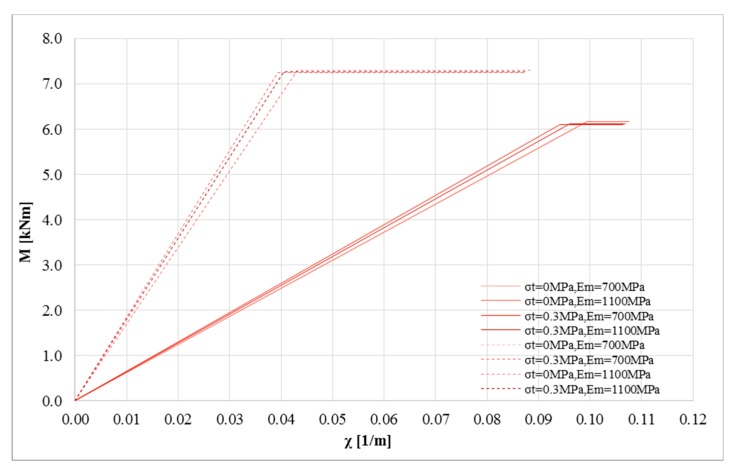
Bi-linearized M-*χ* curves: *P* = 0 N, *σ*_0_ = 1 MPa, *σ_t_* = 0 and 0.3 MPa, *E_m_* = 700 and 1100 MPa for linear (solid lines) and bi-linear (dotted lines) relationships for the strengthening system (basalt fiber).

The ductility estimation can be performed with several approaches, depending on the bending moment-curvature bi-linearization. The minimum curvature ductility of the masonry cross-section strengthened with composites can be calculated as previously clarified (first approach):

(3) $$\mu =\frac{\chi_{u}}{\chi_{y}}$$

where *χ_u_* is the ultimate curvature and *χ_y_* represents the yielding curvature read on the bi-linearized curve. Figure 9 shows the bending moment-curvature diagram bi-linearized according to the first approach. The curves refer to *P* = 0 N, *σ*_0_ = 0 MPa, *σ_t_* = 0 and 0.6 MPa, *E_m_* = 700 and 1100 MPa for linear (solid lines) and bi-linear (dotted lines) relationships for the strengthening system made of inorganic matrix and basalt fiber. The differences in terms of ductility appear clear in this figure. The strengthening system modelled by means of a linear stress-strain curve provides a curvature ductility lower than the bi-linear one.

**Figure 10:**
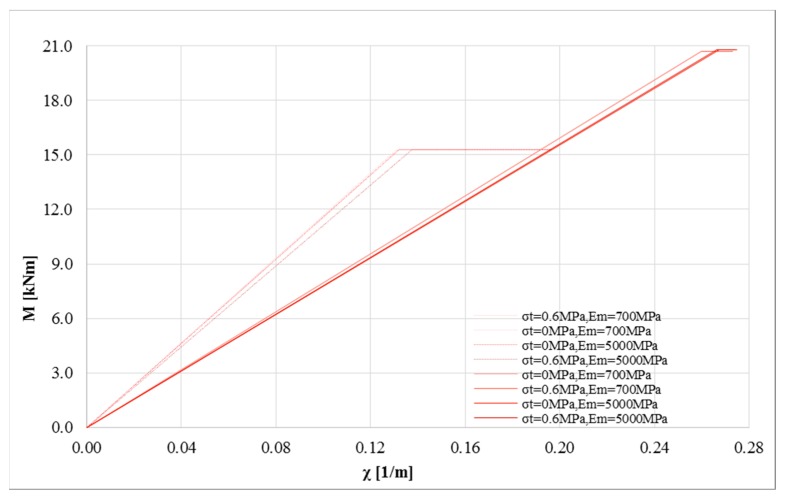
Bi-linearized M-*χ* curves: *P* = 0 N, *σ*_0_ = 8 MPa, *σ_t_* = 0 and 0.6 MPa, *E_m_* = 700 and 5000 MPa for linear (solid lines) and bi-linear (dotted lines) relationships for the strengthening system (basalt fiber).

The same numerical analyses were performed for the second group (Figure 10), where the maximum compressive and tensile strength were increased to 8 and 0.6 MPa, respectively, and the maximum Young’s modulus was fixed equal to 5000 MPa. The great difference in terms of curvature ductility is even more visible.

Therefore, the constitutive laws used to model the composite have a significant impact on both the maximum bending moment value and on the curvature ductility. Therefore, they represent key elements in performing nonlinear analyses.

**Figure 11:**
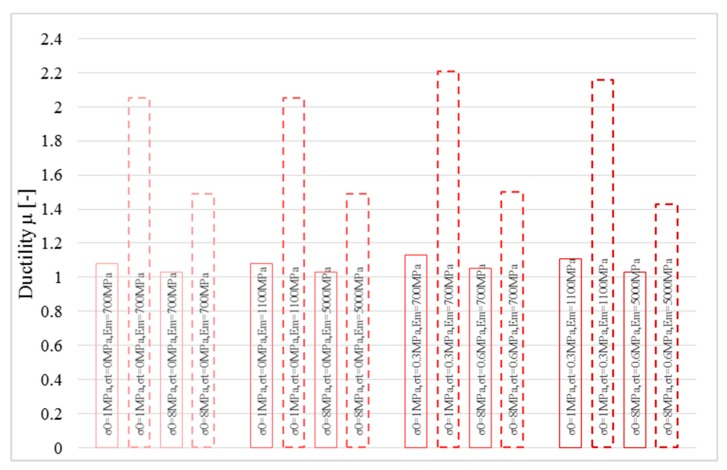
Ductility estimation for the two stress-strain relationships for basalt fiber: *P* = 0 N, for linear (solid lines) and bi-linear (dotted lines).

Finally, the curvature ductility was assessed for the two models as summarized in Figure 11. The strengthening system modelled with a linear stress-strain relationship provides extremely low ductility. Furthermore, with reference to the curvature ductility, the elastic parameters of masonry provide a relevant impact on it.

**Figure 12:**
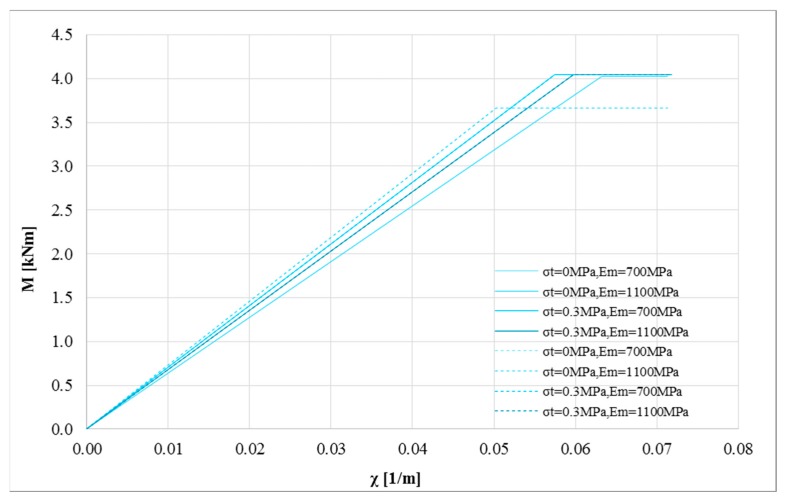
Bi-linearized M-*χ* curves: *P* = 0 N, *σ*_0_ = 1 MPa, *σ_t_* = 0 and 0.3 MPa, *E_m_* = 700 and 1100 MPa for tri-linear (solid lines) and bi-linear (dotted lines) relationships for the strengthening system (hemp fiber).

Similar analysis was performed for the second typology of strengthening system (hemp fiber). Figure 12 shows the bi-linearized curves obtained for masonry with compressive strength *σ_m_* equal to 1 MPa. It is interesting to note that when the strengthening system and the masonry substrate become more compatible, the scatter of the curvature ductility decreases for different mechanical models of the composite. The same effect can be seen for the elastic properties of masonry (Young’s modulus and tensile strength).

**Figure 13:**
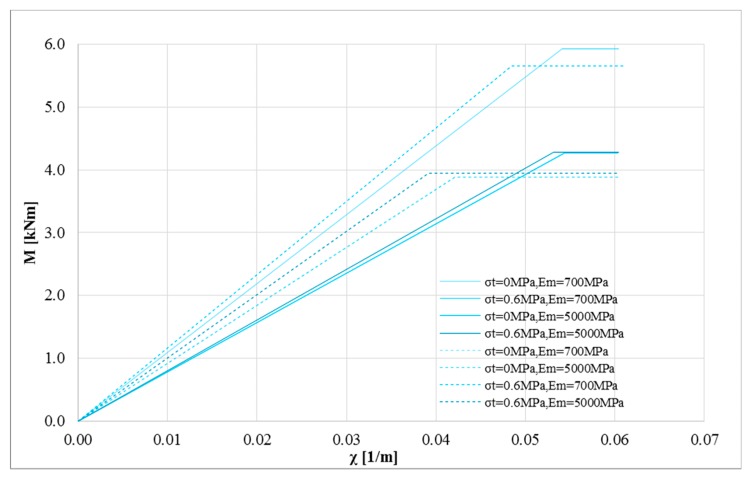
Bi-linearized M-*χ* curves: *P* = 0 N, *σ*_0_ = 8 MPa, *σ_t_* = 0 and 0.6 MPa, *E_m_* = 700 and 5000 MPa for tri-linear (solid lines) and bi-linear (dotted lines) relationships for the strengthening system (hemp fiber).

**Figure 14:**
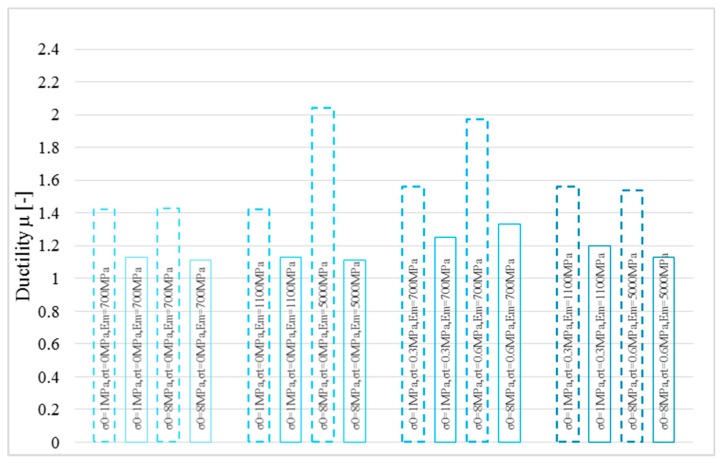
Ductility estimation for the two stress-strain relationships for hemp fiber: *P* = 0 N, for tri-linear (solid lines) and bi-linear (dotted lines).

Figure 13 shows the same analysis performed on masonry with compressive strength equal to 8 MPa. When the compressive strength of masonry *σ_m_* increases, the impact of both the mechanical model of the composite and of the elastic properties of masonry become significant in the ductility estimation. This aspect was clarified in Figure 14, where the ductility estimation is shown.

**Figure 15:**
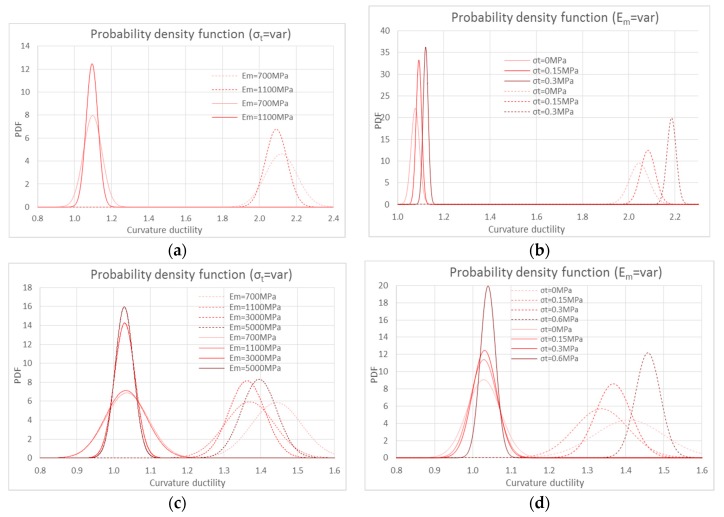
Probability density functions: (**a**,**c**) curvature ductility at different tensile strengths of masonry; (**b**,**d**) curvature ductility at different Young’s moduli, for the two stress-strain relationships for basalt fiber: linear (solid lines) and bi-linear (dotted lines). (**a**,**b**) on weak masonry (*σ*_0_ = 1 MPa) and (**c**,**d**) with *σ*_0_ = 8 MPa.

In order to identify the key aspects, the previously discussed numerical results involve only a few cases of the entire numerical investigation. However, it is interesting to analyze the entire population. In particular, Figure 15 shows the probability density function (PDF) of the curvature ductility for the composite made of synthetic fiber applied on weak masonry (*σ*_0_ =1 MPa). In particular, Figure 15a shows the effect of the tensile strength on the curvature ductility for this strengthening system. It is interesting to note that the linear model (solid lines) shows a lower dispersion compared to the bi-linear (dotted lines). Furthermore, the dispersion decreases when increasing the tensile strength of the masonry. Similarly, Figure 15b shows the effect of the Young’s modulus on the curvature ductility. The linear model (solid lines) shows a lower dispersion compared to the bi-linear one (dotted lines). Furthermore, the dispersion decreases with the Young’s modulus of the masonry.

Figure 15c,d show the same analysis performed on masonry with compressive strength equal to 8 MPa. The Young’s modulus and tensile strength have similar effects on the PDF. It is interesting to note that the difference of the average ductility values decreases when increasing the compressive strength of masonry.

**Figure 16:**
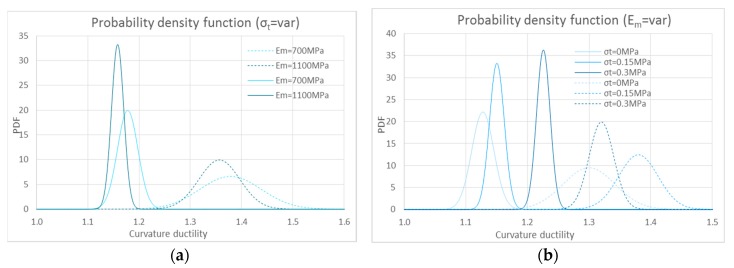

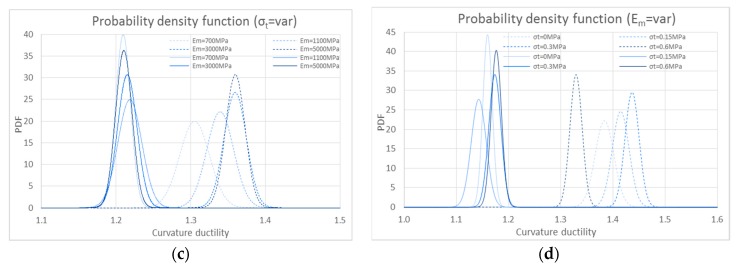
Probability density functions: (**a**,**c**) curvature ductility value to change the tensile strength of masonry; (**b**,**d**) curvature ductility value to change the Young’s modulus, for the two stress strain relationships for hemp fiber—tri-linear (solid lines) and bi-linear (dotted lines). (**a**,**b**) on weak masonry (*σ*_0_ = 1 MPa) and (**c**,**d**) with *σ*_0_ = 8 MPa.

Figure 16 shows the same results for the composite made of natural fiber. An important aspect regards the decrease of the dispersion of numerical results. In particular, the higher the compatibility between composite and masonry substrate, the lower the impact of the stress-strain law of the composite on the ductility estimation. Furthermore, the dispersion between the average values of the curvature ductility also decreases.

## 4. Conclusions

This work focuses on a numerical investigation to assess the impact of stress-strain relationships (i.e., constitutive laws) of several FRCM materials on the flexural behavior of strengthened masonry elements. In particular, the work focuses on strengthening with inorganic matrix and two kinds of fibers (synthetic and natural fibers). The stress-strain curve of masonry was modelled according to the ideal model proposed by Eurocode 6; conversely, the strengthening systems were modelled according to several simplified constitutive laws fitted on the experimental results. When the composite is made of synthetic fiber it can be modelled by means of a bi-linear or linear stress-strain curve, while if a natural material replaces the synthetic fibers, a bi-linear or tri-linear stress-strain model can be adopted.

The strengthening system made of basalt grid is generally characterized by high mechanical performance. Basalt has lower efficiency when applied on weak masonry; therefore, the ultimate condition is due to the local crashing of the masonry. In this case, the mechanical model used to model the composite strongly affects the global behavior of the strengthened masonry element. The elastic properties of the masonry do not influence the structural behavior under a no tension assumption for the masonry. However, if a tensile strength is assumed for masonry, the impact is non-negligible, especially for higher values of the compressive strength of the masonry. The stress-strain curve used to model the composite has an impact on the flexural strength. The numerical results obtained for natural fibers provide similar outcomes, but a first difference involves the higher mechanical compatibility of the strengthening system with the substrate. In this case, the ultimate condition is due to the failure of the composite. On weak masonry, the variability of the structural behavior, changing the elastic properties of masonry, decreases. Furthermore, the stress-strain curve of the composite also has a negligible influence on the estimation of the flexural strength. The bending moment-curvature diagrams show a clear cusp, which occurs when the masonry cross-section cracks, and in some cases, this could limit the capacity of the strengthened masonry.

Finally, the numerical investigation involved the estimation of the curvature ductility according to safe side criteria. The numerical results showed that the stress-strain curves used to model the strengthening system are crucial in the numerical analysis, but the impact reduces when the compressive strength of the masonry increases. However, FRCM behavior strongly influences the curvature ductility at higher masonry compressive strength. The numerical results shown in this work provide a basis for the development of normalized capacity models. They provide key information on the nonlinear assessment and design of masonry elements strengthened with composites.

## Figures and Tables

**Table 1:** Mechanical properties of masonry considered in the numerical investigation.

Compressive strength *σ*_0_ (MPa)	Tensile strength |*σ_t_*| (MPa)	Young’s modulus *E_m_* (MPa)
1, 3, 8	0, 0.15, 0.3, 0.6	700, 1100, 3000, 5000

**Table 2:** Mechanical properties of strengthening systems.

Element	Tensile strength (MPa)	Young’s modulus (MPa)	Ultimate tensile strain (-)
Basalt	1538	71,891	0.0214
Mortar (matrix for basalt)	8	8000	-
Hemp	47	7000	0.00671
Mortar (matrix for hemp)	0.35	8000	-

**Table 3:** Mechanical properties of strengthening systems (homogenized to fiber section): inorganic synthetic fiber-based (I.S.F) and inorganic natural fiber-based (I.N.F.). Both specimens are made of bi-directional fiber grids.

Strengthening system	Young’s modulus *E*_1_^*^ (MPa)	Young’s modulus *E*_2_^*^ (MPa)	Young’s modulus *E_f_* (MPa)	Tensile cracking strength |*σ_m,cr_*| (MPa)	Tensile strength |*σ_cu_*| (MPa)	Initial cracking strain |*ε_m,cr_*| (-)	Final cracking strain |*ε*_2_| (-)	Ultimate strain |*ε_f,u_*| (-)
I.S.F	1,242,982	45,198	71,891	485	1538	0.00039	-	0.0214
I.N.F	346,531	4773	7000	15.16	47	0.000044	0.00217	0.00671

**Figure A1:**
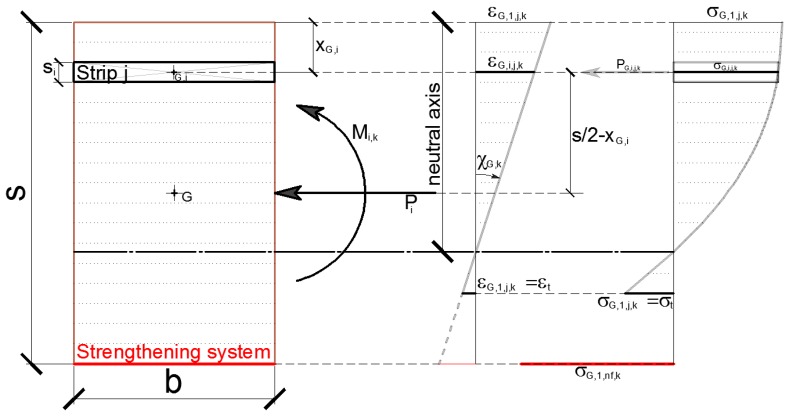
Discrete model to assess the bending moment-curvature diagram.

**Figure A2:**
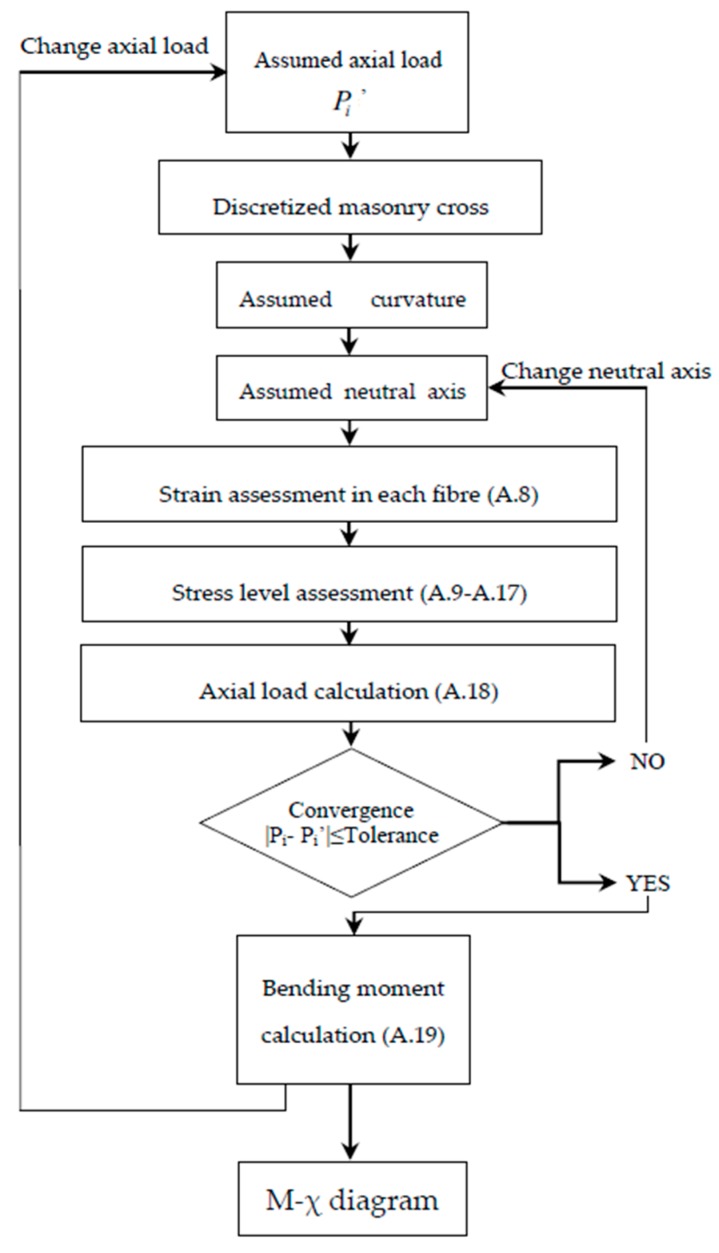
Flowchart of the solver algorithm to assess the bending moment-curvature diagram.

## Appendix A

### Failure Surface of the Strengthened Masonry Section: Solver Algorithm

The ultimate behavior of the masonry cross-section strengthened with composites was assessed starting from the evaluation of the bending moment-curvature diagram. Flexural strength was evaluated for a discrete number of axial load values. The envelope of these points provides the entire failure surface of the strengthened masonry element. Assuming that the strengthening system was applied on one side of the cross-section, it contributes only to one sign of the bending moment (i.e., composite carries loads only if stressed in tension) [18]. When the bending moment puts the strengthening system in compression, it does not contribute and the behavior of the cross-section depends on the masonry only.

The solver algorithm is based on a discrete approach where the cross-section is divided into a finite number of elements (strips). The failure surface depends on the geometry of the cross-section (masonry and strengthening system) and on the mechanical properties of constituents. Furthermore, the accuracy of the numerical approach depends on the discretization level. Upon increasing the discretization level, the solution becomes more accurate, but the duration of the calculation increases. The following section describes the solver algorithm. The input data are:

- Size of the cross-section: *b* and *s*, respectively, width and height;
- Compressive strength of masonry *σ*_0_;
- Tensile strength of masonry through the parameter *α*. It is the ratio between the tensile strength *σ_t_* and the compressive strength *σ*_0_.
  (A1) $$\alpha =\frac{\left|\sigma_{t}\right|}{\sigma_{0}}$$
- Young’s modulus of masonry *E_m_*;
- Multi linear stress-strain curve of strengthening system;
- Cracking stress of matrix *σ_m,cr_*^*^ homogenized to the fiber element;
- Ultimate stress of the composite *σ_c,u_*. It coincides with the tensile strength of the fiber element;
- Initial Young’s modulus *E*_1_^*^ and *E*_2_^*^ of the composite homogenized to the fiber element (for bi-linear and tri-linear models);
- The Young’s modulus of the composite *E_f_* (for linear, bi-linear, and tri-linear models);
- Number of strips, *n_f_*, to discretize the cross-section (the strengthening system is generally modelled as a single strip element);
- Convergence tolerance for the equilibrium equation, generally equal to 1‰ of the axial load *P*;
- Number of steps *n_sP_* to split the axial load range;
- Number of steps *n_sχ_* in order to assess the bending moment-curvature diagram;
- Number of iterations for the solver algorithm. This value is variable and depends on the convergence criteria.

The main parameters that influence the duration of the calculation are the number of steps *n_sP_* and the number of iterations, while the precision of the calculation is due to both the discretization level (i.e., number of strips *n_f_*) and the tolerance imposed. The stress is assumed to be uniformly distributed in the generic strip. A sufficient discretization level ensures that the last assumption is acceptable.

The first information is the maximum number of steps *n_sP_*. This value provides the number of axial loads *P_i_* at which the moment-curvature diagram is assessed. The maximum axial load *P*_0_ is calculated through a pure compression condition:

(A2) $$P_{0}=\sigma_{0}\cdot b\cdot s$$

Under compression stresses, the strengthening system does not react. The calculation was limited to compression only, given the usual conditions for the masonry structures [24,25,26]. The entire range between 0 and *P*_0_ is divided into a number of points *P_i_*:

(A3) $$P_{i}=i\cdot P_{0}/n_{sP}\;\mathrm{for}\;i=0,1,\ldots ,n_{sP}$$

Starting from the bending moment-curvature diagram, the conventional flexural strength *M_rd,i_*(*P_i_*) becomes a point of the failure surface. Once the number of strips *n_f_* is fixed, the cross-section is divided into *n_f_* discrete elements. For each element (i.e., for each strip) the thickness s_f_ and area *A_f_* can be calculated as follows:

(A4) $$s_{f}=s/n_{f}$$

(A5) $$A_{f}=b\cdot s_{f}$$

Another important parameter to implement the solver algorithm is the location of the barycenter of each strip *x_G,j_*. This information is easily found by numbering the strips and starting from the top of the cross-section; the position *x_G,j_* can be easily calculated as follows:

(A6) $$x_{G,j}=\left(n_{j}-1\right)\cdot s_{f}+s_{f}/2\;\mathrm{for}\;j=1,2,\ldots ,n_{f}$$

Finally, the number of steps to assess the bending moment-curvature diagram, *n_sχ_*, must be defined. This value is different from that used for the axial range, *n_sP_*. The last strip, *n_f_*, corresponds to the strengthening system.

The bending moment M and axial load P can be assessed by equilibrium equations of the internal stress diagrams. Therefore, the first step is to assess the strain value achieved in each strip. Under Bernoulli’s assumption, the strain in each strip can be expressed by a linear function. This assumption shall be valid up to collapse, and it allows the assessment of the strain level in each strip. The strain level refers to the barycenter *G_j_* of a generic strip and it depends both on the discretization level *n_f_* and on the step *n_sP_*. The strain level in the generic strip can be calculated starting from the strain value achieved in the top of the cross-section *ε_G,i,j=_*_1,*k*_. In particular, when the neutral axis depth *x_i,k_* is known, this value can be expressed as follows:

(A7) $$\varepsilon_{G,i,1,k}=\chi_{i,k}\cdot x_{i,k}\;\mathrm{for}\;i=1,2,\ldots ,n_{sP}\;\mathrm{and}\;\;k=1,2,\ldots ,n_{s\chi }$$

Finally, the strain level in the generic strip can be expressed as a function of the curvature value *χ_i,k_*:

(A8) $$\varepsilon_{G,i,j,k}=\varepsilon_{G,i,1,k}-\chi_{i,k}\cdot x_{G,j}\;\mathrm{for}\;i=1,2,\ldots ,n_{sP},\;j=1,2,\ldots ,n_{f}\;\mathrm{and}\;k=1,2,\ldots ,n_{s\chi }$$

For a generic strip, the stress value can be calculated from the strain level, according to the mechanical model previously discussed for the masonry:

(A9) $$\sigma_{G,i,j,k}=E_{m}\cdot \varepsilon_{G,i,1,k}\;\mathrm{if}\;\varepsilon_{G,i,j,k}<0$$

(A10) $$\sigma_{G,i,1,k}=\frac{2\cdot \sigma_{0}}{\varepsilon_{0}}\varepsilon_{G,i,1,k}\left(1-\frac{\varepsilon_{G,i,j,k}}{2\cdot \varepsilon_{0}}\right)\;\mathrm{if}\;0\leq \varepsilon_{G,i,j,k}<\varepsilon_{0}\;\mathrm{for}\;i=1,2,\ldots ,n_{sP},\;j=1,2,\ldots ,n_{f}\;\mathrm{and}\;k=1,2,\ldots ,n_{s\chi }$$

(A11) $$\sigma_{G,i,1,k}=\sigma_{0}\;\mathrm{if}\;\varepsilon_{0}\leq \varepsilon_{G,i,j,k}<\varepsilon_{u}$$

and for the strengthening system:

(a) under a tri-linear model assumption:
  (A12) $$\sigma_{G,i,n_{f},k}=E_{1}^{*}\cdot \varepsilon_{G,i,n_{f},k}\;\mathrm{if}\;\varepsilon_{G,i,n_{f},k}\leq \varepsilon_{m,cr}$$
  (A13) $$\sigma_{G,i,n_{f},k}=\sigma_{m,cr}^{*}\;\mathrm{for}\;\varepsilon_{m,cr}<\varepsilon_{G,i,n_{f},k}\leq \varepsilon_{2}$$
  (A14) $$\sigma_{G,i,n_{f},k}=E_{f}\cdot \varepsilon_{G,i,n_{f},k}\;\mathrm{for}\;\varepsilon_{2}<\varepsilon_{G,i,n_{f},k}\leq \varepsilon_{f,u}$$
(b) under bi-linear assumption:
  (A15) $$\sigma_{G,i,n_{f},k}=E_{1}^{*}\cdot \varepsilon_{G,i,n_{f},k}\;\mathrm{if}\;0<\varepsilon_{G,i,n_{f},k}\leq \varepsilon_{m,cr}$$
  (A16) $$\sigma_{G,i,n_{f},k}=E_{2}^{*}\cdot \varepsilon_{G,i,n_{f},k}\;\mathrm{for}\;\varepsilon_{m,cr}<\varepsilon_{G,i,n_{f},k}\leq \varepsilon_{f,u}$$
(c) under linear assumption:
  (A17) $$\sigma_{G,i,n_{f},k}=E_{f}\cdot \varepsilon_{G,i,n_{f},k}\;\mathrm{if}\;0<\varepsilon_{G,i,n_{f},k}\leq \varepsilon_{f,u}$$

The axial load on a generic strip *P_G,i,j,k_* can be calculated from the internal stress level and the axial load on the entire section *P_i_* is as follows:

(A18) $$\begin{array}{c} P_{G,i,j,k}=A_{f}\cdot \sigma_{G,i,j,k}\\ P_{i}=\sum_{j=1}^{n_{f}}P_{G,i,j,k} \end{array}\;\mathrm{for}\;i=1,2,\ldots ,n_{sP},\;j=1,2,\ldots ,n_{f}\;\mathrm{and}\;k=1,2,\ldots ,n_{s\chi }$$

The internal axial load on the cross-section is fixed at each step. Therefore, once it is integrated over the entire cross-section, it does not depend on *k* and *j* indices (i.e., it is not dependent on the curvature *χ* value). The bending moment *M_rd,i,j,k_* can be assessed with a similar approach. In particular, the contribution of the generic strip is provided by the product between the axial load contribution *P_i,j,k_* of the strip and its distance from the center of the cross-section. The bending moment acting on the cross-section *M_i,k_* can be assessed by integrating the previous local bending moment values on the entire section as follows:

(A19) Mi,j,k=PG,i,j,k·(s2−xG,i)Mi,k=∑j=1nfMi,j,k for i=1,2,…,nsP, j=1,2,…,nf and k=1,2,…,nsχ

In this case, the bending moment depends on the curvature (i.e., it depends on the *k* index). The *k* index increases up to the collapse. The ultimate condition can be due to two main failure modes. Starting from a zero curvature value, the stress increases both in the masonry and in the strengthening system. A first threshold is achieved when the masonry cracks:

(A20) $$\sigma_{G,i,n_{f}-1,k}=\sigma_{t}=-\alpha \cdot \sigma_{0}$$

where the expected stress is higher than |*σt*| value, masonry does not carry loads, and stress drops to zero. The tensile strength of masonry provides a negligible contribution when the strengthening system reacts, while it provides a key contribution when the strengthening system is compressed [42,43]. At increasing curvature values, the ultimate condition can be due to one of the three main failure modes:

- crashing of masonry;
- failure of the strengthening system under tensile stress;
- debonding failure mode between the strengthening system and the substrate.

The last failure mode can be easily included as a lower tensile strength of the composite. In particular, for each step *n_sχ_*, the stress achieved in the strengthening system must be compared to the debonding stress value [43]. Otherwise, the calculation continues up to conventional collapse due to the other two failure modes.

It is important to note that other failure modes exist for the strengthened masonry cross-sections. In particular, the cross-section may not react if the compressive strength of the masonry, *σ*_0_, is exceeded. In this case, the strips characterized by a compressive strain higher than the ultimate value do not react and the equilibrium condition is achieved in a cross-section characterized by a reduced height *s*′ < *s*. The curvature increases up to the failure of the strengthening system or up to the loss of equilibrium condition. Figure A1 shows a typical distribution of the internal stresses on a masonry cross-section strengthened with composites.

Starting from zero, curvature can be increased with a fixed step. For a generic step *χ_i,k_*, the axial load *P_i_* is known. The unknown parameter is represented by the neutral axis depth *x_i,k_.* It can be calculated by means of an iterative approach. In particular, once a trial value of the neutral axis *x_i,k’_* is fixed, the strain level in each strip *j* is known. The axial load acting on each strip *P_G_,_i,j,k’_* and on the entire cross-section *P_i,k’_* are easily calculated. The actual neutral axis satisfies the equilibrium equation if there is a convergence on the axial load value with a prefixed tolerance (e.g., 1%).

The next step consists of the evaluation of the bending moment value *M_i,k_* by means of previous equations, and the entire stress level (masonry and composite) can be assessed for each step. The curvature *χ_i,k_* is increased until ultimate condition. In this way, the bending moment-curvature diagram can be assessed. Finally, the bending moment-curvature diagram can be regularized by means of the bi-linearization methods discussed in the paper. Figure A2 shows a schematic flowchart of the previously discussed algorithm.

## Abbreviations

*A_f_*	area of the single strip
*b*	width of the cross-section
*E*_1_^*^	initial Young’s modulus of composite, homogenized to fiber
*E*_2_^*^	second Young’s modulus of the bi-linear model of composite, homogenized to fiber
*E_f_*	Young’s modulus of the fiber
*E_m_*	Young’s modulus of the masonry
*E_mx_*	Young’s modulus of the matrix
*M*	bending moment acting on the cross-section
*M_rd_*	bending strength of the cross-section
*M_rd,i_*	bending strength of the cross-section for an assumed axial load value
*M_rd,i,j,k_*	bending strength of the strip *j* for both assumed axial load and curvature value
*M_i,k_*	bending moment of the cross-section for both assumed axial load and curvature value
*n_f_*	number of strips to discretize the cross-section
*n_sP_*	number of steps to split the axial load range
*n_s_**_χ_*	number of steps to split the bending moment- curvature diagram
*P*	axial load acting on the cross-section
*P*_0_	axial strength of the cross-section
*P_i_*	discrete value of the axial load
*P_G,i,j,k_*	axial load for each strip *j* for both assumed axial load and curvature value
*p*	axial load normalized to the axial strength *P*_0_
*s*	height of the cross-section
*s_f_*	thickness of the single strip
*x_G,j_*	distance of the barycenter of each strip j from the top of the cross-section
*ε*_0_	first plastic strain of masonry
*ε*_2_	final strain of the constant stress-strain curve of the tri-linear model of composite
*ε_c,cr_*	cracking strain of composite
*ε_c,u_*	ultimate strain of composite
*ε_f,u_*	ultimate strain of fiber
*ε_G,j_*	strain level for each strip *j*
*ε_G,i,j_*	strain level for each strip *j* for an assumed axial load value
*ε_G,i,j,k_*	strain level for each strip *j* for both assumed axial load and curvature value
*ε_m,cr_*	cracking strain of mortar
*ε_mu_*	maximum compressive strain of masonry
*μ*	curvature ductility of the cross-section
*σ*_0_	compressive strength of masonry
*σ_c,u_*	tensile strength of composite
*σ_c_*	tensile stress of composite
*σ_f,u_*	tensile strength of fiber
*σ_m,cr_*	cracking strength of mortar
*σ_m,cr_*^*^	cracking strength of mortar, homogenized to fiber
*σ_t_*	tensile strength of masonry
*σ_G,j_*	stress level for each strip *j*
*σ_G,i,j_*	stress level for each strip *j* for an assumed axial load value
*σ_G,i,j,k_*	stress level for each strip *j* for both assumed axial load and curvature value
*α*	tensile strength of masonry, normalized to the compressive strength *σ*_0_
*χ*	curvature value of the strengthened cross-section
*χ**_i,k_*	curvature value for an assumed axial load
*χ**_u_*	ultimate curvature of the bi-linearized curve
*χ**_y_*	yielding curvature of the bi-linearized curve
